# Effect of an infection prevention and control training program on hemodialysis nurses' performance in governmental hospitals in the Gaza Strip: a quasi-experimental study

**DOI:** 10.3389/frhs.2026.1733275

**Published:** 2026-02-03

**Authors:** Suliman Odeh Aladini, Mohamed Kuhail, Mohammed Jebreldar Abuanja Nimer, Susan Ali Zroog, Yousef F. Fahajan, Omar Mohammed Oda Khattab

**Affiliations:** 1 Department of Head of Safety and Infection Prevention and Control, Al Shifa Medical Complex, Gaza, Gaza Strip, Palestine; 2 Department of Clinical Nutrition, Al-Azhar University, Gaza, Gaza Strip, Palestine; 3 Department of Community Health Nursing, International University of Africa (IUA), Khartoum, Sudan; 4 Department of Community Health Nursing, Omar Al-Mukhtar University, Bayda, Libya; 5 Department of Nursing, University College of Applied Sciences, Gaza, Gaza Strip, Palestine; 6 Department of Medical-Surgical Nursing, University College of Applied Sciences, Gaza, Gaza Strip, Palestine

**Keywords:** healthcare-associated infections, hemodialysis nurses, infection prevention and control, nursing performance, training program

## Abstract

**Background:**

Infection prevention and control (IPC) measures are essential in hemodialysis (HD) units because of the increased risk of healthcare-associated infections (HAIs). Nurses play a central role in the implementation of IPC protocols; however, adherence to these guidelines remains inconsistent, particularly in resource-limited settings like the Gaza Strip.

**Objective:**

This study aimed to evaluate the short-term effects (one-week post intervention) of a structured IPC training program on the knowledge and practices of HD nurses working in governmental hospitals within the Gaza Strip.

**Methods:**

A Quasi-experimental design was adopted, utilizing a census sampling technique to include all 112 HD nurses. Participants were assigned to intervention and control groups using a systematic roster-based allocation method. Data were collected via a validated self-structured questionnaire and observational checklist before and one week after training.

**Results:**

Compared with the control group, nurses in the intervention group exhibited statistically significant post-intervention improvements in IPC knowledge (mean percentage score: 79.91 vs. 49.41, *P* = 0.001) and practice (overall adherence: 68.9% vs. 47.6%, *P* = 0.001), representing improvements of 62.68% and 38.63%, respectively.

**Conclusion:**

The IPC training program proved effective in enhancing HD nurses' knowledge and adherence to IPC measures in the short term (one-week post-intervention). However, the study's design limitations and contextual barriers suggest that sustained compliance requires addressing systemic challenges such as resource limitations and high workload. These findings support the need for ongoing structured IPC training in resource-constrained settings.

## Background and justifications of the study

Chronic kidney disease (CKD) is an increasing global health concern, with a prevalence of approximately 9.5% worldwide (1). It has risen from the 25th leading cause of death in 1990 to the 16th leading cause in 2017, and it is projected to become the 5th leading cause of death by 2040 (2, 3). Hemodialysis (HD) is a life sustaining intervention for CKD patients, but HD units carry a high risk for healthcare-associated infections (HAIs) due to frequent vascular access, environmental factors, and patient susceptibility (4). HAIs significantly worsen patient outcomes and are the second leading cause of death (5).

Nurses are central to HD care and are essential for implementing infection prevention and control (IPC) measures (6). However, adherence to IPC guidelines remains inconsistent, particularly in resource-limited settings such as the Gaza Strip (GS). Challenges include limited supplies, high patients loads, instability, and insufficient training, (7–9). Regular training, management support, and supervision are key to increasing knowledge and reducing HAIs and antimicrobial resistance (10, 11).

The number of patients undergoing HD continues to rise, accompanied by high mortality rates compared with those of other diseases. Infection remains the second leading cause of death among HD patients (12, 13). Ensuring patient safety is a key nursing goal, necessitating regular educational interventions to enhance nurses' knowledge and practice in IPC (14). Yet, in GS, many nurses have not received recent IPC training, and systemic barriers hinder sustained adherence. Therefore, this study aimed to evaluate the effect of a structured IPC training program on the knowledge and practices of HD nurses in governmental hospitals in GS, with the goal of informing sustainable strategies to enhance patient safety and care quality in this high-risk, constrained setting.

## Methods and materials

### Study design

This study used a Quasi-experimental design with a census technique to assess the effect of a training program related to IPC guidelines on the performance of HD nurses at governmental hospitals in GS.

### Context of the study

By mid-2024, the global Palestinian population was approximately 14.8 million, with 5.61 million in the State of Palestine. GS, with 2.1 million people, has experienced a 6% population decrease due to conflict-related casualties (15). Ongoing conflicts and blockades have devastated the healthcare system of GS, causing severe shortages and service disruptions and prompting urgent international intervention (16).

### Study setting

The study was conducted at governmental hospitals in the GS, specifically at HD units. These hospitals are Noor Al Kabee, Al-Rantisi Hospital, Al Shifa Medical Complex, Al Aqsa Martyr's Hospital, Hind Aldaghma Center, and Al Najjar Hospital, which face challenges such as limited resources, high patient loads, and geopolitical instability, which affect IPC practices.

### Study period

The study lasted from November 2022 to December 2024 and began immediately after approval from the MoH and the GS Helsinki Committee. The exact study period was almost seven months; however, the extended study period was due to ongoing political instability in Gaza.

### Study population

The study population comprised all registered nurses (*n* = 114) employed in HD units within governmental hospitals in the GS. Eligible participants were those currently working in HD units on morning/afternoon shift, who were available to attend the full duration of the IPC training program, and who provided informed consent to participate in the study. Of these, 112 nurses were included in the final sample, while 2 were excluded due to external leave (*n* = 1) and maternity leave (*n* = 1).

### Sample and sampling

A census sampling technique was employed, including all 112 HD nurses who met the inclusion criteria. A systematic group allocation was performed using the official hospital roster (odd/even ID assignment). Nurses were listed in ascending order of their unique roster ID, and a predetermined systematic allocation rule was applied prior to enrollment to ensure unbiased group assignment; those with odd-numbered IDs were assigned to the control group, and those with even-numbered IDs to the intervention group.

### Data collection tool

The researcher used a self-structured questionnaire and observational checklist, which was based on the Palestinian HD IPC guidelines (17), and the Centers for Disease Control and Prevention checklist (18), to assess HD nurses' knowledge and practices before and after training. The knowledge questionnaire (21 MCQs) was adopted and self-developed based on the Palestinian Ministry of Health Hemodialysis IPC Policies and Procedures Guide (2018). Where the 73-step observational checklist were adopted from Centers for Disease Control and Prevention dialysis IPC (CDC, 2022); the 73 checklist steps are grouped into the eight domains. The English language was used for the questionnaire and checklist because the institutional IPC standards and many of the reference guidelines were in English, and nursing staff in the participating HD units commonly use English for clinical protocols and documentation. An English version of both the questionnaire and the observational checklist has been uploaded as Supplementary File S1.

### Structured questionnaire of knowledge on IPC in hemodialysis procedures

The structured questionnaire, which was administered in English, had two parts: the first included HD nurses' personal details (gender, age, education, and experience), and the second included 21 multiple-choice questions on IPC guidelines related to HD procedures.

### Observational checklist on IPC practices related to hemodialysis

The tool assessed HD nurses' adherence to infection prevention and control (IPC) practices using a 73-step observational checklist. Each item was rated on a three-point Likert scale (0 = not done, 1 = done with remediation needed, 2 = done correctly). For analysis, item scores were summed and converted into a percentage adherence metric using the formula:

OverallIPCpractice(%)=(Totalscore/(73×2))×100.

IPC practice was evaluated across eight domains: (1) arteriovenous fistula/graft cannulation, (2) arteriovenous fistula/graft decannulation, (3) catheter connection, (4) catheter disconnection, (5) catheter exit-site care, (6) hemodialysis injectable medication preparation, (7) hemodialysis injectable medication administration, and (8) routine hemodialysis station disinfection. A total IPC adherence score was obtained by aggregating scores across all domains, resulting in a percentage scale ranging from 0 to 100%. Percentage improvement in practice between pre- and post-intervention assessments was calculated as:

The percentage improvement in practice was calculated using the formula:

[(Post-test%–Pre-test%)/Pre-test%]×100.

All results are reported consistently in percentage adherence. Statistical comparisons were conducted using independent *t*-tests for two-group comparisons and one-way ANOVA for variables with three or more groups, with assumptions checked and reported in the methods. The narrative interpretations are directly linked to the corrected tables, ensuring alignment between reported *p*-values, group differences, and the percentage adherence metric.

### Phase 1: pre-intervention data collection

In the initial phase, the researcher administered a self-structured questionnaire alongside an observational checklist to gather baseline data on participants' sociodemographic characteristics, knowledge, practices, and self-perceived confidence in infection prevention and control (IPC) skills. This data collection occurred before the implementation of the training intervention. To mitigate observation bias: Baseline (Phase 1) data collection took place for one week for each HD unit. To ensure the rigor of practice assessments, several measures were implemented to mitigate potential observation bias. First, standardized observational protocol (the 73-step CDC-based checklist) was followed for all evaluations to ensure consistency and minimize subjective judgment. Second, to reduce the Hawthorne effect, repeated and unannounced visits were interpreted into the data collection schedule, allowing for observation of routine practices rather than performance under scrutiny. Furthermore, the tool's reliability was confirmed during pilot phase, demonstrated strong internal consistency (Cronbach's *α* = 0.921). However, because the same researcher delivered training and conducted assessments, assessor blinding was not possible.

### Phase 2: intervention phase

The intervention involved a two to four-week infection prevention and control (IPC) training program comprising both educational and practical components. To ensure the continuity of hospital operations, the intervention group was divided into smaller subgroups for training. An IPC expert researcher conducted all the sessions, which included interactive lectures, group discussions, and printed handouts aligned with IPC guidelines specific to hemodialysis (HD) units. During the practical component, nurses engaged in hands-on training with real clinical scenarios, where they were required to demonstrate IPC skills individually and repeatedly until each step was performed accurately.

### Phase 3: data collection (posttest one week after intervention)

One week after completion of the training, the researcher reassessed IPC knowledge and practices via the same pretest questionnaire and observational checklist and compared the results with those of the control group to evaluate the effectiveness of training.

### Pilot study

The data collection tools were developed by the researcher based on a comprehensive review of the relevant literature. The tools included (1) a structured questionnaire designed to assess hemodialysis (HD) nurses' knowledge of infection prevention and control (IPC), incorporating demographic data and 21 multiple-choice questions based on IPC guidelines, and (2) a CDC-based observational checklist including 73 steps to evaluate nurses' IPC practices. Content validity was established through expert review by 11 professionals, including HD unit nurses, academic researchers, and IPC specialists. A pilot study was then conducted on 10% of the target sample (12 HD nurses) to evaluate the feasibility, clarity, and applicability of the tools, resulting in minor modifications. Reliability of the study tools was assessed using Cronbach's alpha to evaluate internal consistency. The knowledge questionnaire demonstrated high internal consistency (Cronbach's alpha = 0.899), and the observational checklist likewise showed excellent internal consistency (Cronbach's alpha = 0.921).

### Statistical analysis

Data were analyzed using the Statistical Package for the Social Sciences (SPSS), version 26.0. Descriptive statistics were computed for all variables; categorical variables were summarized using frequencies and percentages, while continuous variables were described using means and standard deviations (SD). For inferential analysis, independent-samples *t*-tests were used to compare mean IPC knowledge and practice scores between the intervention and control groups. One-way analysis of variance (ANOVA) was employed to compare mean scores across more than two categories of sociodemographic variables (e.g., age group, educational level, years of hemodialysis experience). Where applicable, *post-hoc* tests were conducted to identify group differences. All statistical tests were two-tailed, and a *p*-value ≤ 0.05 was considered statistically significant, corresponding to a 95% confidence interval (CI).

## Results and discussion

The study included a total of 112 HD nurses, who were evenly divided into control and intervention groups (*n* = 56 each). The highest proportion of participants were from Al Shifa Hospital (29.5%), followed by Nasser Al Ka'bey and Helal Al Daghma Hospitals (19.6% each). The mean age of the participants was 35.76 ± 7.31 years, with the majority (54.5%) aged between 31 and 40 years. Male nurses comprised 71.4% of the sample, and 78.6% held a bachelor's degree. Most participants (91%) were working in adult HD units, and the average duration of work experience was 5.79 ± 5.12 years. More than half of the nurses (50.9%) reported never having received IPC training, whereas among those who had, 77.7% had received training more than five years prior, highlighting the critical need for updated and regular IPC refresher courses (see Table 1).

**Table 1:** Characteristics of the study participants.

Variable	Control group	Intervention group	Total
	*N* = 56 (50%)	*N* = 56 (50%)	*N* = 112 (100%)
	*N* (%)	*N* (%)	
Work Place			
N. Al Ka'bey	11 (9.8)	11 (9.8)	22 (19.6)
Al Shifa	16 (14.3)	17 (15.2)	33 (29.5)
Al Rantesy	5 (4.5)	5 (4.5)	10 (8.9)
Al Aqsa	8 (7.1)	7 (6.3)	15 (13.4)
H. Al Daghma	11 (9.8)	11 (9.8)	22 (19.6)
Al Najjar	5 (4.5)	5 (4.5)	10 (8.9)
Age (mean 35.76 ± 7.311 years)			
30 years and less	12 (10.7)	18 (16.1)	30 (26.8)
31–40 years	31 (27.7)	30 (26.8)	61 (54.5)
More than 40 years	13 (11.6)	8 (7.1)	21 (18.8)
Gender			
Male	37 (33.0)	43 (38.4)	80 (71.4)
Female	19 (17.0)	13 (11.6)	32 (28.6)
Total	56 (50.0)	56 (50.0)	112 (100.0)
Education level			
Diploma	8 (7.1)	12 (10.7)	20 (17.9)
Bachelor	47 (42.0)	41 (36.6)	88 (78.6)
Master	1 (0.9)	3 (2.7)	4 (3.6)
Type of service			
Adult	51 (45.5)	51 (45.5)	102 (91.0)
Pediatric	5 (4.5)	5 (4.5)	10 (9.0)
Experience (mean 5.79 ± 5.123 years)			
1–5 years	29 (25.9)	29 (25.9)	58 (51.8)
6–10 years	13 (11.6)	23 (20.5)	36 (32.1)
11 years or more	14 (12.5)	4 (3.6)	18 (16.1)
Received previous training			
No	28 (25.0)	29 (25.9)	57 (50.9)
Yes	28 (25.0)	27 (24.1)	55 (49.1)
When received the training? (*N* = 55)			
Less than 1 year	5 (17.8)	6 (22.2)	11 (9.8)
1–5 years ago	2(7.1)	0	2(1.8)
More than 5 years ago	21(75.0)	21(77.7)	42(37.5)

The results in Table 2 reveal significant variations in IPC knowledge and practices across different hospitals, HD units, age groups, genders, education levels, professional experience, and prior training. *post-hoc* Least Significant Difference (LSD) tests, conducted following significant ANOVA results, identified specific pairwise differences among hospitals and education levels. For IPC practice in the intervention group, Al Rantesy Hospital (reference) demonstrated significantly higher adherence compared to Al Shifa (*p* = 0.001), Al Aqsa (*p* = 0.001), and Al Najjar (*p* = 0.001). No significant differences were observed between Al Rantesy and N. Al Ka'bey (*p* = 0.001) or H. Al Daghma (*p* = 0.001). In the control group, Al Najjar Hospital (reference) had significantly lower practice scores compared to all other hospitals (all *p* < 0.05). For IPC knowledge in the intervention group, Al Rantesy (reference) scored significantly higher than N. Al Ka'bey (*p* = 0.217), Al Shifa (*p* = 0.004), Al Aqsa (*p* = 0.016), and Al Najjar (*p* = 0.030), while no significant difference was found compared to H. Al Daghma (*p* = 0.189). Regarding education level, master's degree holders (reference) in the intervention group had significantly higher knowledge scores compared to those with a diploma (*p* = 0.047) or bachelor's degree (*p* = 0.015).

**Table 2:** Differences in performance regarding IPC related to personal characteristics.

Variable	N	Practice Score (Intervention)	Post-hoc (LSD)*	Practice Score (Control)	Post-hoc (LSD)*	Knowledge Score (Intervention)	Post-hoc (LSD)*	Knowledge Score (Control)	Post-hoc (LSD)*
		*Mean* ±* SD*		*Mean* ±* SD*		*Mean* ±* SD*		*Mean* ±* SD*	
Work Place									
N. Al Ka'bey	22	72.20 ± 0.17	0.001*	47.35 ± 0.26	0.011*	81.71 ± 7.79	0.217	37.79 ± 9.76	NA
Al Shifa	33	63.65 ± 0.12	0.001*	46.30 ± 0.31	0.012*	77.59 ± 3.74	0.004*	43.38 ± 8.86	NA
Al Rantesy	10	86.30 ± 0.08	Ref.	65.45 ± 0.21	0.000*	85.00 ± 5.86	Ref.	62.14 ± 18.84	NA
Al Aqsa	15	68.10 ± 0.13	0.001*	47.70 ± 0.17	0.017*	77.89 ± 3.75	0.016*	56.99 ± 51.15	NA
H. Al Daghma	22	69.70 ± 0.12	0.001*	50.05 ± 0.19	0.004*	81.49 ± 3.34	0.189	60.82 ± 5.62	NA
Al Najjar	10	61.60 ± 0.11	0.001*	30.10 ± 0.17	Ref.	78.09 ± 2.46	0.030*	44.28 ± 7.35	NA
*P-value (ANOVA)*		**0.001** *		**0.002** *		**0.028** *		**0.070**	
Type of Services									
Adults	51	67.20 ± 0.15	NA	45.90 ± 0.26	NA	79.41 ± 4.98	NA	48.16 ± 22.36	NA
Pediatric	5	86.30 ± 0.08	NA	65.45 ± 0.21	NA	85.00 ± 5.87	NA	62.14 ± 18.85	NA
*P-value (t-test)*		**0.001** *		**0.183**		**0.022** *		**0.183**	
Age (years)									
≤30	12	67.55 ± 0.16	NA	50.15 ± 1.29	NA	79.10 ± 4.45	NA	47.73 ± 12.99	NA
31–40	31	69.95 ± 0.21	NA	49.60 ± 0.27	NA	81.03 ± 5.69	NA	45.16 ± 11.71	NA
>40	13	68.15 ± 0.10	NA	40.60 ± 0.24	NA	77.53 ± 4.30	NA	61.09 ± 39.76	NA
*P-value (ANOVA)*		**0.657**		**0.112**		**0.181**		**0.091**	
Education Level									
Diploma	8	67.00 ± 0.17	NA	50.90 ± 0.21	NA	80.26 ± 5.69	0.047 *	38.99 ± 13.51	NA
Bachelor	47	68.75 ± 0.17	NA	47.40 ± 0.29	NA	79.30 ± 7.74	0.015 *	51.35 ± 23.28	NA
Master	1	87.95 ± 0.29	NA	32.85 ± 0.00	NA	86.90 ± 7.14	Ref.	41.67 ± 0.00	NA
*P-value (ANOVA)*		**0.112**		**0.459**		**0.049** *		**0.334**	

NA, not applicable; Ref., reference group.

* Independent *t*-test used for 2-group comparisons (Sex, type of service, Previous Training); One-way ANOVA for ≥3 groups (Age, Workplace, Education, Work experience).

Overall, among hospitals, Al Najjar Hospital's control group presented lower IPC practice scores, whereas nurses from Al Rantesy Hospital in the intervention group presented significantly greater IPC knowledge and practices (*P* = 0.001). Pediatric HD nurses consistently outperformed adult HD nurses in both knowledge and practice post-intervention (*P* = 0.022, *P* = 0.001), which is a trend that is consistent with the findings of Mohamed and Fashafsheh (19) and the World Health Organization (20), who attributed this to the high acuity of care in pediatric units. While no significant age-related differences were found in IPC knowledge (*P* = 0.091, *P* = 0.181) or practice (*P* = 0.112, *P* = 0.657), younger nurses scored higher in IPC practice (≤30 years: 50.15 vs. >40 years: 40.60), which aligns with findings by Trifunovic-Koenig, Bushuven, Gerber, Otto, Dettenkofer, Salm and Fischer (21) on adaptability to evolving protocols. Gender differences in IPC adherence were statistically insignificant, although female nurses slightly outperformed male nurses in practice (*P* = 0.253), which is consistent with the findings of Trifunovic-Koenig, Bushuven, Gerber, Otto, Dettenkofer, Salm and Fischer (21) but contradicts the findings of Alsulami, Sacgaca, Pangket, Pasay-An, Al Amoudi, Alreshidi, Alrashedi, Mostoles Jr, Buta and Areola Jr (22), suggesting that external factors such as workload distribution play a role. Education level was a significant determinant of IPC knowledge, with master's degree holders scoring higher than diploma or bachelor-level nurses (*P* = 0.047, *P* = 0.015), yet practice scores did not show a corresponding increase (*P* = 0.112), underscoring the persistent “know-do gap” (23). Professional experience did not significantly impact IPC knowledge (*P* = 0.941, *P* = 0.391) or practice (*P* = 0.211, *P* = 0.603), reinforcing findings from Savul, Ikram, Khan and Khan (24) that structured training, rather than experience, determines compliance. Similarly, prior training had no significant influence on IPC knowledge (*P* = 0.142, *P* = 0.381) or practice (*P* = 0.312, *P* = 0.067), suggesting that one-time training is insufficient for sustained adherence and emphasizing the need for continuous, structured IPC programs (20, 24). The observed variations across hospitals, unit types, and demographic groups highlight the need for IPC training tailored to specific institutional and workforce contexts. Although post-intervention knowledge improved significantly, the persistent knowledge practice gap indicates that systemic barriers such as limited resources, high workload, and institutional policies continue to hinder IPC adherence. These findings support the need for sustained mentorship, periodic refresher training, and stronger policy enforcement to promote long-term IPC compliance across hemodialysis settings.

Table 3 shows that the intervention and control groups were comparable at baseline, with no significant differences in mean knowledge (49.12% vs. 46.61%; *p* = 0.299) or practice adherence (49.70% vs. 48.20%; *p* = 0.560). One week after implementation of the IPC training program, the intervention group demonstrated markedly higher knowledge scores compared with the control group (79.91% vs. 49.40%; *p* = 0.001), reflecting a 62.68% improvement from baseline. A similar pattern was observed for IPC practice, where the intervention group achieved significantly greater adherence than the control group (68.90% vs. 47.65%; *p* = 0.001), corresponding to a 38.63% improvement. Paired analyses confirmed significant pre–post gains only in the intervention group, whereas the control group showed no meaningful change. Overall, these findings confirm that the IPC training program produced substantial and statistically significant short-term improvements in both knowledge and observed practice among hemodialysis nurses.

**Table 3:** Changes in the knowledge and practice of IPC between the two groups.

Knowledge	Phase	Group	N	Mean % score	SD	T	*P* value*
	Pretest—Phase 1	Intervention	56	49.12	12.17	1.044	0.299
		Control	56	46.61	13.23		
	Posttest—Phase 3	Intervention	56	79.91	05.25	9.970	0.001
		Control	56	49.40	22.28		
† The percentage of changes in Knowledge (Interventional Group) is 62.68%							
Practice	Pretest—Phase 1	Intervention	56	49.70	13.65	0.584	0.560
		Control	56	48.20	13.35		
	Posttest– Phase 3	Intervention	56	68.90	08.90	9.630	0.001
		Control	56	47.65	13.90		
† The percentage of changes in practice (Interventional Group) is 38.63%							

* Independent *t*-tests compare intervention and control groups at each time point. Paired *t*-tests evaluate pre- to post-intervention change within each group.

Despite these gains, certain practices, such as routine disinfection, showed only marginal improvement, likely reflecting persistent challenges such as limited resources and high patient-to-nurse ratios. These findings are consistent with prior research by Tabash, Kashkash and Eljedi (25) and Eljedi and Dalo (9), who reported similar post-training improvements in IPC adherence among Palestinian HD nurses. Additionally, the results align with those of Singh, Kaur, Saini, Singh, Aggarwal and Chandra (26), who reported a 50% increase in IPC compliance following training interventions in northern India.

Noteworthy improvements were observed in specific clinical procedures, including arteriovenous fistula/graft cannulation (*P* = 0.001) and catheter disconnection care (*P* = 0.001), supporting the results of Tannous, El-Saed, Ameer, Khalaf, Mohammad, Molaeb and Alshamrani (27) and Kadium (28). However, other domains, such as medication administration (*P* = 0.393) and linen management (*P* = 0.234), showed minimal gains, suggesting the need for supplementary strategies beyond training, such as workflow optimization and strict policy enforcement, to achieve sustained IPC compliance. This is supported by Tu, Elling, Behnke, Tseka, Kafanikhale, Mofolo, Hoffman and Cronk (29), who emphasized that nurses in high-pressure environments may prioritize task efficiency over protocol adherence, despite adequate training.

Overall, the study shows that hands-on IPC training improves knowledge and practical skills; however, sustained compliance requires addressing systemic barriers through ongoing education, simulated practice, and structured feedback to reinforce behavior change.

## Limitations of the study

This study has several limitations. First, although the study included a control group, and pre-post measurement, the allocation was non-random and based on a systemic roster method. Formal allocation concealment was not implemented, as the researcher was aware of group assignment during recruitment, which represents a common limitation of quasi-experimental studies conducted in operational clinical settings. Second, assessor blinding was not feasible, as the same researcher delivered training and conducted evaluations, though standardized protocols, repeated unannounced visits, and a tool with established reliability (Cronbach's *α* = 0.921) to mitigate observation bias. Inter-rater reliability statistics (e.g., ICC or Kappa) were not calculated, as observations were conducted by a single trained assessor. Third, the short post-intervention assessment period limits conclusions regarding long-term retention of IPC knowledge and practices. Finally, contextual systemic barriers such as limited resources, high patient-to-nurse ratios, and operational disruptions in the Gaza Strip may affect the sustainability of improved practices. Future studies would benefit using centralized randomization systems, blinded independent assessors, and longer follow-up periods to strengthen causal inference and evaluate sustained compliance.

## Conclusion and recommendations

The Quasi-experimental study demonstrates that a structured IPC training program led to significant improvement in HD nurses' knowledge and practices in governmental hospitals in the Gaza Strip. These short-term gains underscore the value of targeted educational interventions in resource constrained setting. However, considering the study's limitations, sustained IPC compliance will require more than training alone and must address the persistent systemic barriers highlighted in this context. To translate these improvements into practice change, we recommend: (1) implementing regular and mandatory IPC training for HD nurses, (2) integrating IPC into nursing curricula, (3) ensuring consistent availability of essential IPC supplies, (4) fostering multidisciplinary collaboration, and (5) enforcing IPC guidelines through continuous supervision and clear accountability measures. Future research should employ longer follow-up periods and blinded assessment to better evaluate the sustainability of training effects.

## Authors' contributions

SOA and OMOK supported tool development, training implementation, hospital coordination, and data collection. MK conceptualized and led the study. MJAN supervised the methodology, and contributed to data analysis and interpretation. SAZ contributed to data analysis and interpretation. YFF provided statistical support and assisted in manuscript preparation. All authors reviewed and approved the final manuscript.

## Data Availability

The original contributions presented in the study are included in the article/supplementary material, further inquiries can be directed to the corresponding author.
