# Supplementary material for: Effect of an infection prevention and control training program on hemodialysis nurses' performance in governmental hospitals in the Gaza Strip: a quasi-experimental study
Source: Front Health Serv. 2026 Feb 3;6:1733275. doi: 10.3389/frhs.2026.1733275 (PMC12909495; doi:10.3389/frhs.2026.1733275)
Supplement: Supplementary file 1 [file Datasheet1.pdf]

<u>Part I: Sociodemographic data</u>

This section classifies your personal data. Please read the terms carefully and answer them by placing a mark (√) in front of the item that represents your case.

Work place :---

Age (years): --

Gender: ☐ Male ☐ Female

Marital status:

☐ Married ☐ Single ☐ Divorced ☐ Widow

Educational level:

☐ Diploma ☐ Bachelor ☐ Master

Services offered in the facility

☐ **Adults** ☐ **Pediatric** ☐ **Both**

Years of Experience in hemodialysis unit : ------------------------------

Have you formally received training on infection control and prevention guideline in hemodialysis ?

☐ Yes ☐ No

If yes, How long ago?

☐ Less than 1 year ☐ 1 – 5 years ☐ More than 5 years

Part II: Nurse's Knowledge about Infection Control and Prevention in hemodialysis unit.

Select all correct answers in each question. If you can't understand the language/terminology inform the researcher for interpretation verbally.

1. Source of knowledge about Infection control and prevention in Hemodialysis unit is/are:

a. University .
b. In-service .
c. Conference.
d. Workshop .

2. Infection control and prevention ICP is defined as ?

a- A practical, evidence-based approach preventing patients and health workers from being harmed by avoidable infections.
b- The discipline concerned with preventing healthcare-associated infections;
c- A type of procedure that is carried out with the intent of preventing the transmission of communicable diseases by assessing, planning, implementing, and evaluating infection-control policies.
d- A-scientific approach and practical solution designed to prevent harm caused by infection to patients and health workers.

3.Who is at risk of infection in Hemodialysis unit?

a. Heath care worker.
b. Patient.
c. Visitors .
d. Community.

4. Hand Hygiene is Recommended :

a- Before and after patient contact.
b- After touching patients surrounding s
c- Before aseptic technique
d- After body fluid exposure risk.

5. The strategies to Control Health Care Association Infection are:

a. Eliminate of the causative agent .
b. Reservoir control .
c. Control Portal of entry and exit.
d. Mode of transmission cycle break.

6. Standard Precaution include which of the following steps?

a. Hand hygiene and personal protective equipment.
b. Item processing and disinfection.
c. Environmental cleaning and waste management.
d. Respiratory hygiene and cough etiquette.

7. What are the steps to take if you are exposed to <u>Needle Stick Injury or Blood Splashes?</u>

a. Wash needle sticks and cuts with soap and water.
b. Do not squeeze the site of injury.
c. Report the incident and Immediately seek medical treatment.
d. Flush splashes to the nose, mouth, or skin with water.

8. Which of the following PPE should be worn before insertion <u>Central Line Catheter</u> ?

a. Sterile gown.
b. Sterile gloves.
c. Mask.
d. Eye protection, cap.

9. Which of the following skin antiseptic used before Central Line Insertion in hemodialysis patient?

a. Chlorhexidine 2%.
b. Povidone iodine 10%.
c. Chlorhexidine 0.5% + alcohol 70%.
d. Alcohol 70%.

10- Which of the following True statement about hemodialysis machine disinfection?

a. The dialysis machines must be disinfected by thermal disinfection at the end of each day, by carrying out a complete cycle at a temperature of 80 C for 30 minutes.
b. The machines must be disinfected between patient, by chemical disinfection according to manufacture recommendation.
c. A low-level disinfectant can be used to clean soiled machine surfaces
d. The disinfected surfaces should remain wet, then air drying to allow for sufficient contact time.

11. Which of the following statement related to Safe Injection Practice ?

a. Prepare injections using aseptic technique in a clean area.
b. Disinfect the rubber septum on a medication vial with alcohol before piercing.
c. Do not use needles or syringes for more than one patient.
d. Do not combine the leftover contents of single-use vials for later use.

12-Which of the following example of Disinfectant?

a. Alcohol 70%.
b. Glutaraldehyde.
c. Ortho-phthalaldehyde.
d. Hydrogen peroxide.

13-Contact precaution are important to prevent transmission of :

a. Clostridium difficile (C. diff).
b. MRSA (Methicillin Resistance Staff. aures).
c. VRE (Vancomycin resistance Enterococci).
d. CRE (Carbapenem Resistance Enterobacteriaceae**).**

14. Which of the following essential steps when Arteriovenous fistula/graft Cannulation?

a. Apply skin antiseptic, and allow it to dry.
b. Performed hand hygiene, Put on new clean gloves.
c. Clean site with soap and water.
d. Insert needle and connect to blood lines aseptically**.**

15.**Which of the following lab investigation should done to the patient in the first visiting to hemodialysis unit ?**

a. HBsAg.
b. anti -HBc total .
c. anti-HBs .
d. anti-HCV.

16. At the end of Hemodialysis ,Machine Surface should be disinfected by

a. Chlorine500-1000ppm .
b. Alcohol 70%.
c. Survinous.
d. Quaternary ammonium compounds

17-Which of the following is true regarding Respiratory Hygiene and Cough Etiquette ?

a. Cough and sneeze on disposable napkins and disposed in no-touch receptacles then wash your hand.
b. Cough and sneeze over shoulders if napkins not available.
c. Keep distance of 3 feet from others when coughing .
d. Cover your mouth and nose when coughing or sneezing.

18- Linen and Textiles Management include which of the following statement ?

a. Contaminated linen and Textiles should be carefully removed with a minimum of agitation.
b. Should not be sorted or prerinsed in patient care areas.
c. Containing soiled items in a laundry bag or designated bin.
d. Bags should sturdy enough (leak-resistant),and not fill to more than two third full.

19- Management of Medical and sharp waste include which of the following step ?

a. Should be collected in plastic or water proof Container.
b. Closed and collected when full for 2/3 or every 7 days .
c. Used needles and syringes are discarded into safety box.
d. Separated according to color code bags.

20- Which of following Correct and safe procedures in the process of cleaning up blood spills and body fluids?

a. Control access to area around the spill
b. Use absorbent disinfectant granules or peds to contain the spill
c. Put on appropriate personal protective equipment
d. Remove the contaminated waste using the scoop and scroper and carefully dispose of waste into infectious waste bag

21-**What are High touch Surfaces , that need frequent cleaning ?**

a. Doorknobs
b. Bedside work table
c. Hand washing faucet handle
d. Hemodialysis (HD) machine keyboard

Part III: infection control and prevention practice in hemodialysis unit checklist

Infection Control and Prevention Practice Observation Checklist				
**The Candidate did the following**	**Mark**	**Done correctly**	**Done incorrectly**	**Not done**
**Arteriovenous Fistula/Graft Cannulation Observation:**				
Site cleaned with soap and water				
Hand hygiene performed (staff)				
New, clean gloves worn				
Skin antiseptic applied appropriately				
Skin antiseptic allowed to dry				
No contact with fistula /graft site (after antiseptics)				
Cannulation performed aseptically				
Connect blood lines aseptically				
Gloves removed				
hand hygiene performed				
**Arteriovenous Fistula/Graft Deccannulation Observation :**				
Hand hygiene performed				
New ,clean gloves worn				
Disconnect from blood line aseptically				
Needles removed aseptically				
Clean gloves worn (patient /staff) to compress site				
Clean gauze /bandage applied to site				

If other activities performed between needle removed , hand hygiene is performed and new ,clean gloves are worn				
Staff gloves removed				
Staff hand hygiene performed				
Patient gloves removed and hand hygiene performed (if applicable)				
**Catheter Connection Observation**				
Mask worn properly (if required)				
Hand hygiene performed				
New, clean gloves worn				
Catheter hup scrubbed				
Hub antiseptic allowed to dry				
Catheter connected to blood lines aseptically				
Gloves removed				
Hand hygiene performed				
**Catheter Disconnection Observation**				
Mask worn properly (if required)				
Hand hygiene performed				
New ,clean gloves worn				
Catheter removed from blood line aseptically				
Catheter hup scrubbed				
Hub antiseptic allowed to dry				
New caps attached aseptically (after disconnection)				
Gloves removed				
Hand hygiene performed				
**Catheter Exit Site Care Observation**				
Mask worn properly (if required)				
Hand hygiene performed				
New ,clean gloves worn				
Skin antiseptic applied appropriately				
skin antiseptic allowed to dry				
No contact with exit site (after antisepsis)				
Antimicrobial ointment applied				
Dressing applied aseptically				

Gloves removed				
Hand hygiene performed				

The candidate did the following	Mark	Done correctly	Done incorrectly	Not done
**Hemodialysis injectable medication preparation**				
Medication preparation done in designated area				
Medication preparation area is clean				
All vials are inspected				
Hand hygiene performed				
Septum of all vials disinfected				
All vials entered with new needle are new syringe				
Medication preparation done aseptically				
All single dose vials discarded				
All multi dose vials discarded or stored properly				
**Hemodialysis injectable medication administration**				
Medication properly transported to patient station				
Hand hygiene performed				
Clean gloves worn				
Injection port disinfected with antiseptics				
Medication administered aseptically				
Syringes discarded at point of use				
Gloves removed				
Hand hygiene performed				
**Hemodialysis station routine disinfection observation**				
All supplies removed from station and prime bucket emptied				
Gloves removed ,hand hygiene performed				
Station is empty before disinfection initiated				
New clean gloves worn				
Disinfection applied to all surfaces and prime bucket				
All surfaces are wet with disinfection				
All surfaces allowed to dry				
Gloves removed and hand hygiene performed				
No supplies or patient brought to station until disinfection complete				

Thank You for Your Participation
